# Thoughts on War

**Published:** 1917-04

**Authors:** 


### THOUGHTS ON WAR

### Personal Preparedness.

In view of the disturbed political conditions:

Avoid all speculative investments.

Limit investments to real estate ownership and first mortgages or to things that you can actually use or closely supervise.

Keep an adequate reserve in the bank and, in an emergency, of actual cash, preferably in gold.

So far as possible, do business on a cash basis both ways.

Lay in a reasonable supply of all non-perishable necessities of life, including drugs and chemicals, etc., whenever obtainable at normal prices but do not overdo this precautionary measure.

Do not fool away either time or money. Even philanthropies should now be of practical nature.

Dispose of "unfinished business," financial, social, professional, scientific, literary and personal, including your will.

### Health Insurance.

In view of the probable increase of the military establishment of the country and the possible control of many forms of industry, necessarily including the medical supervision and care of a large part of the population, this time is particularly inappropriate for the adoption of any radical experiment of socializing the medical profession. Without regard to this point, there are two weak spots in all such measures: the arbitrary classification of labor and lack of protection for the special form of labor (medical) to be utilized. Medical organizations generally should adopt a concerted and definitely formulated policy that all schemes for utilizing the labor of the medical profession shall, in a practical way, guarantee the proper balance of work and pay for the labor employed. It is entirely conceivable that not only the fairest and most efficient solution of the various problems involved, but the most convenient, will be the extension of the duties of the established government medical services, probably with some degree of transfer of authority from nation to state. War or even extensive military preparation will automatically provide medical care for a considerable proportion of the industrial population, more extensively and more thoroughly than in the past. If the general need of medical attendance as provided in typic health insurance bills exists, it can be carried out by medical officers in time of peace but of preparation of war so as to allow the economic utilization of an adequate medical corps. During actual hostilities, if there be such on our territory, it can be carried out as in other wars by systematic philanthropy, without burdening the state in a time of emergency, with the distribution of a further burden called by another name. If we have war, its cessation will cause more hardship to medical officers suddenly thrown out of employment than to any other part of the population. By carrying on the principles of health insurance by an existing, well trained medical corps, the various problems can be solved economically and efficiently.

### The Sublime and the Ridiculous in Patriotism.

A true story of patriotism is as follows: A man entered a recruiting station in England, bared his abdomen and took off a shoe and stocking and said, "Now, will you take me?" Rejected on account of a hernia and some form of talipes, he had submitted to radical operations in order further to risk his life for his country. This story deeply impressed an audience, moving some to tears. One hearer merely grinned. When upbraided for his cynicism, he explained that he had been doing somewhat the same thing himself but the circumstances were just different enough to take out the heroism and appeal to his sense of the ridiculous. He had been having his teeth filled so that, if anything did happen, he would be ready.

After all, it is just as practical from the standpoint of patriotism and applicable to a far greater number of cases, to have the teeth in good condition, to be vaccinated against small pox and typhoid, to make sure that vision is normal or corrected by lenses, to cut down superfluous flesh and build up muscle by exercise, to get rid of adenoids, nasal obstructions and piles, as to show the heriosm of the British applicant. The medical profession can do much toward preparedness by reducing the physical unfitness due to very minor conditions.

### The Attitude Toward Certain Means of Warfare.

At several medical demonstrations, we have been impressed with the instinctive antipathy toward gas bombs, liquid fire, etc. Men viewed with strictly professonal interest or, at most, with expressions of sympathy for the sufferer, the most horrible and painful lesions produced by missiles of various kinds but destruction of life by gases and lesions produced by intentional and direct application of fire, even if not involving any more pain than the former and even if resulting in less permanent disability, produced expressions of anger, by no means dependent on natural prejudices for or against either side in the conflict. During the Civil War, a physician seriously proposed the dissemination of small pox virus in the south. The need of greater offensive efficiency was, at the time, especially urgent and probably from the individual standpoint, most persons would choose an attack of small pox to a severe wound by any ordinary weapon of war, but the proposal was received with a degree of popular indignation which threatened the safety of the proposer. We admit the inability properly to analyse these instinctive prejudices and sentiments but we feel that they very literally represent the principle Vox populi, vox Dei and we urge that while actual conflict is still only a remote possibility, they be crystalized into a decision as to public policy in the event of war, that shall not be changed under any provocation. When, at the close of the first conflict of the Revolution, Mr. Emerson's hired man emerged from a place of safe hiding and despatched a wounded British soldier with a hatchet, he made a blot on American history that nothing can obliterate. While we are still capable of thinking calmly, let us impress it on our own minds and on those not able to think logically for themselves, that wars last a very short time in comparison with the periods of peace in which we have ample time for reflection and let us insist on a uniform policy of humane warfare before war even becomes probable.

### Neutrality.

So far as can be judged from the conduct of audiences at picture shows and the like, and from newspaper discussions, the people of the United States have sincerely and, in the main, successfully, endeavored to preserve a genuine neutrality during the war. There have, of course, been individual opinions, some directly due to nationality of birth, some obviously influenced by hereditary loyalty to one or another of the ancestral countries, some formed by dispassionate judgment so far as the individual has been aware. That the last class of sympathies have been genuinely due to reasoning is well shown by the fact that they have in some instances been contrary to suppositious hereditary loyalty, while in other instances, they have followed ancestral affiliations when the mere fact of being an American was due ultimately to bad treatment by the mother or ancestral country. The fact that the judgments have differed irrespective of heredity, shows that, while a large number of decisions must be incorrect, they are in the aggregate due to logic and not to prejudice.

Recent developments have made even the theory of absolute impartiality of sympathy, incompatible with immediate national duty. But, in a broader sense, neutrality should still be preserved. Patriotism to our own country should by no means imply racial prejudice or hatred. Neither should we single out for our hostility any individual or group of individuals who happen to personify to us, a nation or group of nations. Perhaps we should go even farther than this. The present contest is viewed by many as one between democratic and autocratic systems of government but we must not forget that the theoretic form of government does not necessarily indicate its essential qualities nor do the people necessarily choose in accordance with our own notions. Nor must we ignore the fact that autocracy has been directed to produce prosperity and efficiency, honest and economic municipal government, and a degree of personal liberty in peace which in many respects are far ahead of what our own democracy has produced.

**Time to Quit Frittering Away Time and Money.**

Individually and collectively, the time is ripe to turn needless wastes into resources that may be needed for war and that would greatly increase the general prosperity in peace. Alcohol, tobacco and chewing gum are not the only things whose cost might be applied to building battle ships. Eliminating blotting paper, calendars and circulars would build a whole fleet and leave much paper for more useful purposes. Not even Uncle Sam through the post office nor the rag man make enough to count from this waste. It is estimated that this country spends 72 millions a year on church music. The mere official work, printing and postage of "good causes" charitable, educational, literary, scientific, etc., must mount into the millions, not to mention the many times larger contributions for the real work of such organizations. Much of this work is necessary, from the humanitarian standpoint; a good deal of it is desirable, much of it is merely playing at being either philanthropic, literary or scientific. Practically all of the really useful work is carried on either by organizations that duplicate or multiply one another's efforts or that duplicate similar lines of activity by various branches of the government. It is safe to say that half of the outlay of the people on this sort of activity is wasted—the total certainly running into hundreds of millions.

Right here and now, there is complaint of high prices and a whirl wind state investigation holds out no hope of relief except further economy of the kind of going without ordinary comforts. Yet a retail dealer in food stuffs reports his business stagnant because the people will not pay what he has to charge, the wholesale market amply stocked and jobbers throwing out vegetables and fruit daily rather than sell the whole at a lower price. For more than a year after the war began, food prices in England, France and Germany were, with some explainable exceptions, actually lower, not only than our own present prices but lower than those current in America for several years.

The U. S. Government, in 1914, considered and decided against a cent a gallon tax on gasoline. Private corporations have gradually added a tax of more than 10 cents. True, it is estimated that we are threatened with an exhaustion of the supply, but in about 25 years, with many possible sources of supply to hear from and ample leisure to work out the problem of a substitute fuel or source of energy.

There are only a few A B C's of economics. It is time that as a people, we master at least the whole primer. If necessary, we can carry on quite a good sized war on what we waste.

